# Physical Performance During the Menopausal Transition and the Role of Physical Activity

**DOI:** 10.1093/gerona/glaa292

**Published:** 2020-11-24

**Authors:** Dmitriy Bondarev, Taija Finni, Katja Kokko, Urho M Kujala, Pauliina Aukee, Vuokko Kovanen, Eija K Laakkonen, Sarianna Sipilä

**Affiliations:** 1 Gerontology Research Center and Faculty of Sport and Health Sciences, University of Jyväskylä, Finland; 2 Neuromuscular Research Center, Faculty of Sport and Health Sciences, University of Jyväskylä, Finland; 3 Faculty of Sport and Health Sciences, University of Jyväskylä, Finland; 4 Department of Obstetrics and Gynecology, Pelvic Floor Research and Therapy Unit, Central Finland Central Hospital, Jyväskylä, Finland

**Keywords:** Longitudinal changes, Menopause, Muscle power, Muscle strength, Walking

## Abstract

**Background:**

To examine longitudinal changes in physical performance during the menopausal transition and the role of physical activity (PA) in these changes.

**Methods:**

Based on follicle-stimulating hormone levels and bleeding diaries, women (47–55 years) were classified as early (*n* = 89) and late perimenopausal (*n* = 143) and followed prospectively until postmenopausal status, with mean duration of 17.5 and 13.8 months, respectively. Physical performance was measured by handgrip force, knee extension torque, vertical jumping height, maximal walking speed, and 6-minute walking distance. Physical activity was self-reported and categorized as inactive, low, medium, and high. Longitudinal associations of menopausal status, physical performance, and related changes with PA level were analyzed using generalized estimation equations adjusted for duration of hormonal therapy.

**Results:**

A significant decline over the menopausal transition in handgrip force (−2.1%, 95% CI −3.8 to −0.4), knee extension torque (−2.6%, 95% CI −4.5 to −0.8), and vertical jumping height (−2.6%, 95% CI −4.2 to −1.1) and a significant increase in 6-minute walking distance (2.1%, 95% CI 1.4 to 2.7) were observed in the total sample. A significant interaction of PA by time was observed in handgrip force and in vertical jumping height. High PA women had greater increase in handgrip strength but greater decline in vertical jumping height than medium, low, and inactive women (all *p* ≤ .001).

**Conclusions:**

Both early and late perimenopausal women show decline in muscle strength and power during the transition to postmenopause. Physical activity seems to influence physical performance during the menopausal transition but understanding the benefits of PA requires interventional studies.

Physical performance is essential for the maintenance of everyday functional capacity. Age-related decline in muscle strength and power are known causes of low walking speed, mobility limitations, and disabilities among older people (1). The rate of decline typically accelerates in the later decades of life; however, in women, this accelerated pattern may begin already in midlife due to hormonal changes occurring during the menopausal years (2,3).

The menopause, defined as the final menstruation, occurs on average between the ages of 46 and 52 depending on the population studied and signifies the end of a woman’s reproductive years (4). Due to the ovarian failure, significant changes in the hypothalamo-hypophyseal control system become noticeable several years before menopause. This leads to a decline in a serum estrogen concentrations together with an elevation in follicle-stimulating hormone (FSH) levels (5). With the recent advancement in life expectancy, a significant proportion of women live in the postmenopausal state, with its accompanying physiology and pathophysiology, for almost one-third of their lives.

Female sex steroids play an important role in maintaining skeletal muscle homeostasis and function (6,7). We and others have shown that postmenopausal women have lower muscle mass, muscle strength, and muscle power than premenopausal women (8–11). However, no association between muscle strength and the menopausal transition stages has also been reported (12,13).

Only a few longitudinal studies have investigated the associations of the menopausal transition with physical performance. One such study investigated changes over 3 years from the premenopausal to postmenopausal stage and found a decline in pinch strength but not in handgrip strength (14). Another study showed a decline in handgrip strength, sit-to-stand time, and preferred walking speed in pre- and perimenopausal women followed up to postmenopause over a 5-year period (15). It is difficult to draw conclusions based on the existing studies, as they have reported only a limited number of physical performance measurements. Moreover, the conflicting results obtained for some measures of physical performance may be due to the use of self-reports on the menstrual cycle as the sole method of establishing menopausal status, as this protocol may in some cases lead to misclassification of menopausal status (16).

Physical activity (PA) is a significant factor in the physical functioning of middle-aged women (17,18). In our recent paper, we showed that a higher level of PA was associated with better physical performance and muscle mass in 47- to 55-year-old pre-, peri-, and postmenopausal women (8,11), indicating that higher levels of PA may, at least partially, slow down the loss of physical performance during the menopausal transition. However, at present, the complex interrelationship between the menopausal transition, physical performance, and PA has not been verified with longitudinal observations.

This longitudinal study thus extends the previously reported cross-sectional results on differences in physical performance at different menopausal stages (8), using data drawn from the population-based Estrogenic Regulation of Muscle Apoptosis (ERMA) cohort study (19). In the present study, menopausal status was assessed with bleeding diaries and hormonal measurements, allowing us to follow-up perimenopausal women prospectively until they became postmenopausal, an approach which provides a unique opportunity to examine changes in physical performance close to menopause and thereby reduce possible confounding factors. Thus, the aim of the study was (i) to assess changes in physical performance during the menopausal transition from the peri- to postmenopausal state utilizing a comprehensive set of measurements to assess physical performance, and (ii) to investigate whether these changes vary with the level of PA.

## Method

### Study Design and Participants

**Figure 1.**
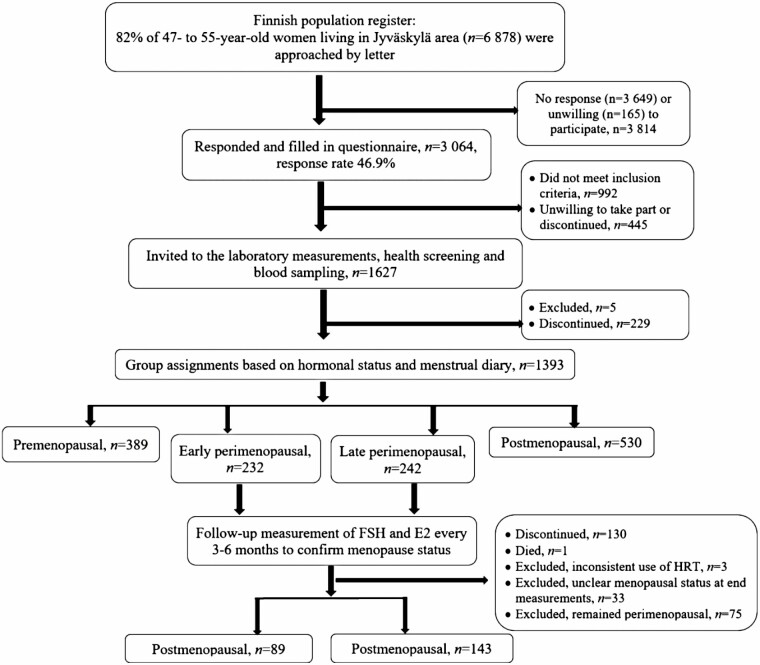
Recruitment of participants.

This study forms a part of the ERMA study (19). The present study flow chart is shown in Figure 1. Women aged 47–55 years living in the city of Jyväskylä and neighboring municipalities were randomly selected from the Finnish National Registry maintained by the Population Register Centre. An invitation to participate in the study was sent to 6878 potential participants. Women who self-reported a body mass index of >35 kg/m^2^, being currently pregnant or lactating, having medical conditions affecting ovarian function (such as bilateral ovariectomy), users of estrogen-containing hormonal therapy (HT) or other medications affecting ovarian function, or having health concerns or conditions that may affect skeletal muscle function or preclude them from participating in daily physical activities were excluded from the study. The response rate to the postal invitation to participate in the study was 47%. Eligible participants (*n* = 1627) were invited to the laboratory and 1393 women categorized as premenopausal, early perimenopausal, late perimenopausal, or postmenopausal based on self-reported menstrual bleeding and measured FSH levels. Of these, 232 women in the early perimenopausal stage and 242 women in the late perimenopausal stage were followed up until they reached postmenopause. During the follow-up, all participants kept a menstrual diary and gave blood samples: FSH and 17β-estradiol levels were measured approximately every 6 months in the early perimenopausal women and every 3 months in the late perimenopausal women until their postmenopausal status was confirmed. The analysis in the present study comprises women whose performance of at least one of the physical performance measurements was acceptable in early or late perimenopause and after the follow-up when postmenopausal (*n* = 232).

All study participants gave their written informed consent. The study was conducted in accordance with the guidelines on good clinical and scientific practice laid down by the Declaration of Helsinki and was approved by the ethics committee of the Central Finland Health Care District (KSSHP Dnro 8U/2014).

### Measurements

#### Demographics, anthropometry, and body composition

Data on level of education, marital status, presence of musculoskeletal diseases or conditions, current and past smoking, use of a progestogen-releasing intrauterine coil, or other progestogen preparation (for contraception or to treat a gynecological bleeding disorder) were self-reported with a structured questionnaire. Age was calculated from date of birth to the date of the characterization of menopausal status.

*Body mass* was measured with a digital scale and *height* with a stadiometer. *Body mass index* was calculated as body mass (kg) divided by height squared (m^2^).

### Menopausal Status

Menopausal status was determined based on the analysis of bleeding diaries and serum concentrations of FSH following the Stages of Reproductive Aging Workshop (STRAW) criteria (20). A complete description of the grouping by menopausal status has been reported earlier (19). In brief, participants with FSH 17–25 IU/L were assigned to the early perimenopausal group and participants with FSH 25–30 IU/L were assigned to the late perimenopausal group. In addition, participants with irregular menstrual cycle were classified as early perimenopausal if their FSH was 9.5–25 IU/L. Participants reporting occasional bleedings that had occurred during past 3 months were classified as late perimenopausal even though their FSH value might exceed 30 IU/L (cutoff value for postmenopause).

*Postmenopausal status* was considered reached if no menstrual bleeding during the past 6 months was reported and FSH > 30 IU/L in 2 consecutive measurements. Participants with FSH > 30 IU/L during follow-up and no menstrual bleeding during the past 6 months were invited for check-up within a month of the last measurement, and if FSH remained >30 IU/L and no bleeding had occurred, were considered to be postmenopausal and invited to the laboratory to repeat the baseline muscle performance measurements. Follow-up time was calculated as the time between the initial hormonal measurements and the time when postmenopausal status was confirmed. The participants who started HT during the follow-up were invited to the physical performance measurements 6 months after starting HT.

### Serum Hormone Measurements

Systemic FSH and 17β-estradiol levels were measured from fasting serum samples collected between 8:00 and 10:00 am. At the baseline measurements, if a woman was in her menstrual cycle, the data were collected during cycle days 1–5. For serum separation, blood samples were centrifuged for 10 minutes at 2.200*g*. Follicle-stimulating hormone and 17β-estradiol level were measured with an IMMULITE 2000 XPi analyzer (Siemens Healthcare Diagnostics, Camberley, United Kingdom) using solid-phase, enzyme-labeled chemiluminescent competitive immunoassay. According to manufacturer’s instructions FSH was determined using a monoclonal murine anti-FSH. The analytical sensitivity limit provided by the manufacture is 0.1 IU/L. Interassay coefficient of variation for the lower end of detection range is 2.9% with a precision value 6.8 ± 0.2 IU/L (mean ± *SD*); and for the higher end of the detection range is 3.9% with precision value 103 ± 3.2 IU/L. Accurate reportable range for E2 is 0.02–2 nmol/L.

The FSH and 17β-estradiol follow-up measurements were performed in 2 consecutive visits (control and final follow-up) and averaged to minimize the effect of daily fluctuations. Participants who started using HT after the baseline measurements were assessed only during the final follow-up visit.

### Physical Activity

Physical activity was assessed by questionnaire on a 7-point scale (21) ranging from household chores to competitive sports and with items assessing leisure-time PA pattern. The instrument has previously been validated in middle-aged women (22).

The response options were: (1) I do not move more than is necessary in my daily routines; (2) I go for casual walks and engage in light outdoor recreation 1–2 times a week; (3) I go for casual walks and engage in light outdoor recreation several times a week; (4) Once or twice a week, I engage in brisk physical activity (eg, yard work, walking, cycling) that causes some shortness of breath and sweating; (5) Several times a week (3–5), I engage in brisk physical activity (eg, yard work, walking, cycling) that causes some shortness of breath and sweating; (6) I exercise several times a week in a way that induces rather strong shortness of breath and sweating during the activity; (7) I do competitive sports and maintain my fitness through regular training. As options 1, 2, 3, and 7 received only 1, 4, 11, and 1 responses, respectively, we combined and recoded them as follows: inactive (options 1, 2, 3), low PA (option 4), medium PA (option 5), and high PA (options 6 and 7).

### Physical Performance

A detailed description of the measurement of physical performance has been given earlier (8). All measurements of physical performance were conducted at baseline and follow-up with the same procedure and equipment. Briefly, *maximal isometric knee extension force* was measured in a sitting position from the side of the dominant hand on a custom-made dynamometer chair (Good Strength; Metitur Oy, Palokka, Finland) with a knee angle set at 60° from full extension. Participants were encouraged to extend the knee to produce maximal force. To calculate *maximal isometric muscle torque*, the best attempt measured in Newtons (N) was multiplied by lower leg (lever arm) length (the distance between the lateral joint line of the knee and the midline of a cuff around the ankle).

*Handgrip force* on the dominant side was measured in Newtons with the elbow flexed at an angle of 90° and arm fixed to the armrest of the dynamometer chair. Participants were instructed to grip the handle as forcefully as possible. Grip was maintained for 2–3 seconds and the peak value taken for analysis.

*Lower body muscle power* was assessed by a countermovement jump on a contact mat. The countermovement jump describes the ability to elevate the body’s center of gravity as fast as possible during a vertical jump (vertical jumping height). Vertical jumping height was calculated in cm from flight time (*t*): (g × *t*^2^) ÷ 8 × 100 (23).

In all the above tests, 3–5 maximal attempts were performed and best performance taken as the result.

*Maximum walking speed* was assessed over 10 m in a laboratory corridor and walking time measured with photocells. Participants were encouraged to “walk as fast as they could.” The best time of 2 trials was taken as the result.

*Aerobic capacity* was assessed by the 6-minute walking test. The test was conducted on a 20-m indoor track, and participants were instructed to complete as many laps as they could during 6 minutes. The distance covered in meters was used in the analysis.

Data on demographics, height, body mass index, and PA were obtained at the same time as the baseline measurements.

### Data Analysis

Participant characteristics are shown as means and standard deviations or as percentages. The normality of distributions was tested with the Shapiro–Wilk test. Discrete baseline characteristics between the early and late perimenopausal groups were compared by chi-square tests or, in the case of continuous variables, by independent samples *t* test. For non-normally distributed data, comparison was performed with the Mann–Whitney *U* test.

The general linear model with the interaction of perimenopausal group by time was used to evaluate the level of significance in changes in physical performance during the menopausal transition. The analysis was adjusted by the duration of HT (days; entered in the model as a mean-centered variable). Mean percentage changes in physical performance variables were calculated as [(follow-up − baseline)/baseline × 100].

To investigate the role of PA in the link between menopausal status and physical performance, we used the general linear model for repeated measures data with the interaction of baseline PA level group by time. Tukey test was used for post hoc comparisons between groups.

A total of 37 women started HT during the follow-up. Therefore, the analyses were adjusted for the duration (days) of HT (entered in the model as a mean-centered variable). Because HT can influence neuromuscular function (24,25), and thus affect physical performance, we also conducted sensitivity analyses solely with the women (*n* = 196) who did not use HT (either estrogen-containing or progestogen-containing medications) during the follow-up (Supplementary Tables 1 and 2).

Due to high individual variation, changes in 17β-estradiol and FSH levels over the follow-up were calculated as the absolute differences between the baseline and follow-up measurements. These values were used in the correlation analysis (Pearson *r*) to see if the changes in the hormonal levels were associated with changes in physical performance. The correlation analysis was conducted for a subsample of women, excluding those who were using progestogen medication at baseline and those who started HT during the follow-up.

All analyses were performed with R, version 3.5.1. For significant chi-square values, a post hoc analysis was conducted using the fifer package for R. For repeated measures data the analysis was performed using package *ez* (to extract ANOVA values). Packages *geepack* and *emmeans* were used to calculate marginal means and respective confidence intervals for different groups across time points. All tests were performed at an a priori significance level of .05.

## Results

Participants’ demographic and health characteristics at baseline are shown in Table 1. The late perimenopausal women were slightly older than the early perimenopausal women. The level of 17β-estradiol was significantly higher and level of FSH significantly lower in the early peri- than late perimenopausal women. No between-group differences were observed in the other demographic or health characteristics.

**Table 1.** Demographics, Health, and Gynecology at Baseline

Variables	Early Perimenopausal, *n* = 89	Late Perimenopausal, *n* = 143	Chi-Square Test, *p* Values
Age, years	51.19 (1.98)	51.85 (1.82)	.010 (*t* test)
Education, *n* (%)	*n* = 88	*n* = 143	.516
Primary	1 (1)	5 (3)	
Secondary	16 (18)	28(20)	
Tertiary	71 (81)	110 (77)	
Marital status, *n* (%)	*n* = 88	*n* = 143	.813
Single	11 (13)	14 (9)	
Married or registered partnership	65 (74)	109 (77)	
Divorced, separated, or widowed	12 (13)	20 (14)	
Smoking, *n* (%)	*n* = 88	*n* = 142	.110
Never	65 (73)	94 (66)	
Former	21 (25)	35 (25)	
Current	2 (2)	13 (9)	
Musculoskeletal problems, *n* (%)	*n* = 87 32 (37)	*n* = 142 49 (34)	.727
Use of hormonal contraception, *n* (%)	*n* = 88	*n* = 143	.170
Nonuser	47 (52)	89 (62)	
Former user	10 (12)	20 (14)	
Current user*	31 (36)	34 (24)	
Using progestogens, *n* (%)	*n* = 88	*n* = 142	.384
No	62 (70)	108 (76)	
Former	19 (22)	21 (15)	
Current	7 (8)	13 (9)	
Physical activity, *n* (%)	*n* = 88	*n* = 143	.562
Inactive	13 (15)	18 (13)	
Low	21 (24)	34 (23)	
Medium	36 (41)	70 (49)	
High	18 (20)	21 (15)	
Body mass index, kg/m^2^	25.35 (4.17)	25.70 (3.85)	.517 (*t* test)
17β-estradiol, nmol/L	0.46 (0.34)	0.25 (0.17)	**<.001** (*t* test)
Follicle-stimulating hormone, IU/L	18.29 (4.99)	47.02 (20.65)	**<.001** (*t* test)

*Notes*: *Current users of estrogen-containing medication (given orally or transdermally) were excluded from this study; therefore, the category “Current user” only includes users of progestogen-containing medications. Values in bold indicate statistically significant results.

Mean duration of follow-up was 17.6 (*SD* 8.5) months for the early peri- and 13.8 (*SD* 8.6) months for the late perimenopausal group (*p* < .001). A total of 40 women started HT during the follow-up from which 3 women were excluded due to the inconsistent reporting of HT use. Thus, 21 women in the early perimenopausal group and 16 women in the late perimenopausal group who started HT were included in the main analysis. The duration of HT varied from 2 to 337 days. The HT starters were significantly younger (50.6 years, *SD* 1.8), than those who did not start HT (51.8 years, *SD* 1.8, *p* = .001). There were no statistically significant differences at baseline between the HT starters and those who did not use HT in education (*p* = .418), marital status (*p* = .661), smoking (*p* = .706), musculoskeletal problems (*p* = .470), body mass index (*p* = .989), progestogen medication use (*p* = .385), or PA level (*p* = .228). There were also no significant differences between HT starters and nonstarters at baseline in 17β-estradiol level (*p* = .540) or in FSH level (*p* = .173).

In the early perimenopausal women, the level of FSH increased significantly from 18.3 (*SD* 5.0) to 64.8 (*SD* 30.7) IU/L (2.44, *SD* 3.47 IU/L per month) and in the late perimenopausal women significantly from 47.0 (*SD* 20.6) to 71.1 (*SD* 30.8) IU/L (1.69, *SD* 3.73 IU/L per month). The level of E2 declined significantly from 0.46 (*SD* 0.34) to 0.23 (*SD* 0.41) nmol/L (0.011, *SD* 0.04 IU/L per month) in the early perimenopausal women. In the late perimenopausal women, the level of E2 declined significantly from 0.25 (*SD* 0.17) to 0.21 (*SD* 0.31) nmol/L (0.002, *SD* 0.02 IU/L per month).

Table 2 shows the results of the physical performance tests for the whole sample and separately for the early and late perimenopausal groups. A significant decline over time was observed in hand grip strength (−2%), knee extension torque (−3%), and vertical jumping height (−3%) and a significant increase in 6-minute walking distance (2%). No significant interaction of perimenopausal group by time was observed in any of the physical performance variables.

**Table 2.** Changes in Physical Performance During the Menopausal Transition (time) in the Early and Late Perimenopausal Groups (groups)

Variables, Group, *n*	Time		Change, % (95% CI)	ANOVA, *p* Values		
	Baseline, Mean (95% CI)	Post, Mean (95% CI)				
				Time	Group	Time × Group
Hand grip (N)						
Total sample, *n* = 230	314 (306; 322)	305 (297; 313)	**−2.1 (−3.8; −0.4)**	**.002**	.681	.100
Early peri, *n* = 89	319 (307; 331)	306 (295; 318)	**−3.5 (−6.0; −1.1)**			
Late peri, *n* = 141	310 (300; 319)	304 (294; 315)	−1.5 (−3.8; 0.8)			
Maximum knee extension torque (Nm)						
Total sample, 193	155 (151; 160)	150 (145; 155)	**−2.6 (−4.5; −0.8)**	**<.001**	.810	.273
Early peri, *n* = 76	156 (149; 163)	149 (142; 157)	**−4.1 (−6.5; −1.6)**			
Late peri, *n* = 117	155 (148; 161)	151 (145; 157)	−1.6 (−4.2; 0.9)			
Vertical jumping height (cm)						
Total sample, *n* = 208	18.8 (18.3; 18.4)	18.2 (17.6; 18.8)	**−2.6 (−4.2; −1.1)**	**<.001**	.897	.241
Early peri, *n* = 83	18.9 (18.1; 19.8)	18.1 (17.3; 19.0)	**−3.9 (−6.3; −1.6)**			
Late peri, *n* = 125	18.7 (18.8; 19.3)	18.2 (17.6; 18.9)	**−2.2 (−4.2; −0.3)**			
Maximum walking speed (m/s)						
Total sample, *n* = 221	2.65 (2.55; 2.68)	2.63 (2.57; 2.69)	1.1 (−0.2; 2.40)	.656	.949	.420
Early peri, *n* = 87	2.62 (2.52; 2.73)	2.62 (2.53; 2.71)	0.1 (−1.5; 3.2)			
Late peri, *n* = 134	2.61 (2.53; 2.69)	2.64 (2.56; 2.71)	1.3 (−0.2; 2.9)			
Six-minute walking test (m)						
Total sample, *n* = 205	665 (656; 673)	667 (668; 686)	**2.1 (1.4; 2.7)**	**<.001**	.209	.178
Early peri, *n* = 81	672 (659; 686)	682 (667; 696)	**1.5 (0.4; 2.70)**			
Late peri, *n* = 124	657 (646; 668)	673 (661; 684)	**2.5 (1.6; 3.3)**			

*Notes*: CI = confidence interval. All analyses adjusted for the duration of hormonal therapy (HT) use. Values in bold indicate statistically significant results.

Similar results were obtained when the women who were using progesterone medication at baseline and those who started HT during the follow-up were excluded from the analysis (Supplementary Table 1).

The correlation coefficients between changes in 17β-estradiol and FSH levels over the follow-up and changes in physical performance varied between *r* = 0.100–0.024 (*p* = .163–.757) for 17β-estradiol and *r* = 0.117–0.001 (*p* = .102–.993) for FSH.

Table 3 shows changes in the physical performance measures in relation to baseline PA level. A significant interaction of PA group by time was observed in handgrip force and vertical jumping height. In handgrip force a significant increase was observed in women with high PA, whereas a significant decline was observed in women with medium PA. Post hoc comparisons showed that, for the high PA women change in handgrip strength was significantly greater than for medium PA (*p* = .001), low PA (*p* < .001), or inactive group (*p* < .001). No significant differences in change in handgrip strength were observed when comparing medium PA group with low PA (*p* = .355) and inactive group (*p* = .151) or when comparing low PA group with inactive PA group (*p* = .527).

**Table 3.** Changes in Physical Performance During the Menopausal Transition (time) as a Function of Physical Activity (PA; groups)

PA, Groups, *n*	Time		Change, % (95% CI)	ANOVA, *p* Values		
	Baseline, Mean (95% CI)	Post, Mean (95% CI)		Time	Group	Time × Groups
Hand grip (N)				.16	**<.001**	**<.001**
Inactive, *n* = 31	290 (272; 309)	292 (279; 304)	3.9 (−2.0; 9.8)			
Low PA, *n* = 54	302 (288; 316)	294 (279; 310)	−1.9 (−5.3; 1.5)			
Medium PA, *n* = 104	318 (307; 329)	300 (288; 312)	**−5.6 (−7.9; −3.3)**			
High PA, *n* = 38	337 (320: 354)	345 (326; 365)	**3.4 (0.2; 6.7)**			
Maximum knee extension torque (Nm)				**<.001**	.190	.060
Inactive, *n* = 24	148 (135; 161)	141 (130; 153)	−2.6 (−9.7; 4.5)			
Low PA*, n* = 45	148 (138; 157)	149 (138; 158)	0.9 (−2.3; 4.1)			
Medium PA, *n* = 91	157 (150; 164)	152 (145; 159)	**−3.1 (−5.7; −0.5**)			
High PA, *n* = 30	166 (155: 177)	156 (144; 168)	**−6.0 (−9.6; −2.2)**			
Vertical jumping height (cm)				**.004**	**<.001**	**.003**
Inactive, *n* = 25	17.0 (15.7; 18.2)	17.2 (15.8;18.6)	−1.0 (−4.4; 6.4)			
Low PA*, n* = 49	18.4 (17.4; 19.3)	18.2 (17.1; 19.2)	−0.9 (−4.2; 2.2)			
Medium PA, *n* = 90	18.4 (17.7; 19.2)	17.6 (16.9; 18.4)	**−3.5 (−5.4; −1.6**)			
High PA, *n* = 36	21.5 (20.2: 22.8)	20.5 (19.1; 21.8)	**−4.0 (−7.4; −0.7)**			
Maximum walking speed (m/s)				.563	.226	.752
Inactive, *n* = 28	2.55 (2.38; 2.71)	2.59 (2.43; 2.76)	2.4 (−0.4; 5.3)			
Low PA, *n* = 52	2.61 (2.49; 2.73)	2.61 (2.51; 2.72)	0.9 (−1.7; 1.4)			
Medium PA, *n* = 100	2.58 (2.48; 2.68)	2.60 (2.51; 2.69)	1.2 (−0.9; 3.4)			
High PA, *n* = 38	2.77 (2.62: 2.92)	2.75 (2.61; 2.89)	0.3 (−2.4; 3.0)			
Six-minute walking test (m)				**<.001**	**<.001**	.150
Inactive, *n* = 25	641 (622; 660)	650 (635; 664)	1.4 (−0.6; 3.5)			
Low PA, *n* = 50	650 (635; 664)	664 (651; 677)	**2.3 (1.1; 3.5)**			
Medium PA, *n* = 93	660 (648; 673)	678 (663; 692)	**2.6 (1.5; 3.7)**			
High PA, *n* = 34	707 (685: 728)	709 (688; 730)	0.8 (−0.8; 2.5)			

*Notes*: CI = confidence interval. All analyses adjusted for the duration of hormonal therapy (HT) use. Values in bold indicate statistically significant results.

In vertical jumping height a significant decline was observed in women with medium and high PA, whereas physically inactive and women with low PA showed no changes. For high PA group the decline in vertical jumping height was significantly greater than for medium PA (*p* < .001), low PA (*p* = .001), or inactive group (*p* < .001). There were no differences in change in vertical jumping height when comparing medium PA group with low PA (*p* = .822) and inactive group (*p* = .232). Comparison of low PA group with inactive PA group also showed no differences in changes in vertical jumping height (*p* = .207).

No significant interactions of PA group by time were observed in knee extension torque and walking performance. A significant group effect was, however, observed in 6-minute walking distance: postmenopausal women with high PA had higher values than postmenopausal women who were inactive or had low PA (Table 3). Similar results were obtained when the women who were using progesterone medication at baseline and those who started HT during the follow-up were excluded from the analysis (Supplementary Table 2).

## Discussion

We observed an average of 2%–3% decline in physical performance from early perimenopause to postmenopause. No changes were observed in walking speed, while distance travelled in 6 minutes had significantly improved at postmenopause. When analyzing the early and late perimenopausal groups separately, the decline in muscle strength was significant only in the early perimenopausal group, whereas in muscle power, both groups showed a significant decline during the menopausal transition. Women reporting a high level of PA showed a significant increase in handgrip strength but a greater decline in lower limb muscle strength and power than the medium, low, or inactive physically active women following the menopausal transition.

Although differences between pre- and postmenopausal women in muscle strength and power have been observed in cross-sectional studies (8,10), only a few longitudinal studies have investigated changes in physical performance during the menopausal transition (14,15). These studies showed a decline in handgrip strength in women transitioning from pre- to postmenopause over 3 and 5 years. The one study that provided measurements for components of physical performance other than grip strength found a nonsignificant postmenopausal decline in lower limb muscle strength but a significant decline in preferred walking speed and sit-to-stand time (15), contrary to our findings. Our study showed a significant decline in both lower limb muscle strength and power and no change or even an improvement in walking parameters.

The decline observed in muscle strength and power can most likely be attributed to wide-ranging alterations in hormones related to the menopausal transition. A meta-analysis showed that women using estrogen or combined estrogen-progesterone therapy displayed better muscle strength than those not using these therapies (26). This suggests that female steroids play an important role in muscle function. Estrogen receptors are expressed at multiple sites along the neuromuscular system (27), and thus estrogen may act in preserving of muscle mass and quality, for example, through apoptotic pathways (6) as well as through neural factors involved in muscle strength generation, such as neural drive and motor coordination (27). Our recent study with the ERMA participants showed mean appendicular muscle mass to be 4% lower in the postmenopausal than premenopausal women (11), suggesting that a loss of muscle mass around menopause may be one of the reasons for the decline in muscle strength.

The present analysis also showed a 2%–4% decline in muscle power that was significant in both early and late perimenopaual groups. Maintenance of muscle power is a crucial factor for functional independence and may be more important than muscle strength for active participation in daily activities (28). As power generation is based on muscle force and contraction velocity, both can be influenced by the menopausal transition. One of the candidates for the decline in muscle power is a mechanism in the central nervous system that governs the speed of motor unit recruitment. It has recently been demonstrated that rapid force development depends on the speed of recruitment and rate of discharge of motor neurons (29). There is also evidence that in the early follicular phase, when estrogen is low in comparison to the high-estrogen phases, motor unit discharge rates (30) and voluntary activation (31) are lower. These may be indicative of changes in excitability in the central nervous system during the menopausal transition.

Performance in walking speed and distance travelled in 6 minutes were not affected by the menopausal transition. In fact, we even saw an improvement in distance travelled during 6 minutes. Supporting our findings, the Montreal-Ottawa New Emerging Team (MONET) study found no changes in maximal oxygen consumption during the menopausal transition (32). In another study, however, postmenopausal women showed reduced exercise tolerance and impairment in maximal aerobic function as compared with age-matched premenopausal women (33). The improvement in walking distance, observed in our study, may be related to motivational factors. For healthy middle-aged women, the test is clearly submaximal as, unlike running or performing maximal strength and power measurements, it does not require maximum effort. This may give participants some latitude for improvement, especially if they remember their baseline results. On the other hand, there could also be a ceiling effect which may obscure discrimination in the functional performance between the 2 time points.

It is difficult to explicitly speculate the clinical relevance of our findings. Only very few studies have investigated the rate of the decline in muscle strength and the clinical outcomes and none, on our knowledge, among middle-aged women. One study among women aged >65 reported a 3-year decline of 1 kg in knee extension strength among those who developed incident mobility disability and that of 0.6 kg among those who did not (34). We observed an average decline of 0.7 kg among early perimenopausal group already after a relative short (an average of 1.5 years) follow-up period. This indicates that the rate of decline in muscle strength and power observed during the menopausal transition needs to be further investigated as a clinical sign in physical functioning decline.

Physical activity has been shown to be an independent determinant of physical performance in middle-aged women (18,35). For handgrip strength, women with high PA showed an increase in performance, whereas for lower limb strength and power we observed that women with a high PA level showed a greater rate of decline than those with a lower PA levels during the menopausal transition. In handgrip strength, the women with a medium PA level showed the highest rate of decline. Although the greater decline in lower limb strength and power among high physically active women may be due to regression toward the mean, this may also invite speculation as to whether the menopausal transition has a greater effect on women with high PA than women with low PA. Exercise intervention studies with predominantly postmenopausal women have shown that exercise is associated with a lower estrogen level (36), and thus women with high PA may show a greater decline in their hormonal level during the menopausal transition; this in turn may negatively affect muscle strength. In one study, women with moderate PA had a higher estradiol level than woman with low or high PA, suggesting a U-shaped relation between estrogen and PA (37). It is thus possible that high PA may disrupt the hormonal profile in perimenopausal women, especially when exercise is associated with low energy intake (38). This raises the question of the optimal dosage and timing of PA for women with diverse histories of PA during the menopausal transition.

Although we assumed that changes in serum sex steroids drive the changes in muscle strength, we did not observe significant correlations between changes in 17β-estradiol and FSH and changes in physical performance. Previous studies on this relationship have predominantly investigated postmenopausal women using HT (24,35,36). These studies reported conflicting results due to diversity in the type, dose, and duration of the exogenous hormones prescribed. The menopausal transition is a time of instability in several hormones (eg, FSH, estradiol, inhibin, progesterone), and thus it may be that no single hormonal change explains the decline in muscle strength.

This study has its limitations. Since our study did not include premenopausal women, our sample comprised women with a relatively short menopausal transition time (mean time to menopause of only 1.3 years during the follow-up). Despite the series of FSH measurements some women may have been categorized as postmenopausal too early due to fluctuations in hormone levels during the menopausal transition and due to the requirement of no menstrual bleeding over 6 months.

Physical activity may have changed during the follow-up and thus it may have differently influenced participants’ physical performance. In the present study and with the methods used we could not track precisely at which point and for how long time during the follow-up our participants may have changed their level of PA. Therefore, the only baseline PA level was used in the models and PA during the follow-up period was not included in the models.

Moreover, PA was self-reported and included only leisure-time PA. This excludes occupational activities and may also exclude occasional light- and moderate-intensity PA. Self-reports are also subject to response bias. However, self-reported information on leisure PA may be more reliable than, for example, accelerometer-based measurements. The PA levels captured by our questionnaire have shown not especially high, but statistically significant correlations with accelerometer-measured moderate-to-vigorous leisure-time PA and both correlated equally with the physical performance measurements (22). Our analysis was limited to healthy and nonseverely obese participants, a choice which restricts the generalizability of the results to other populations.

Among the strengths of the study is the longitudinal follow-up design and that we were able to comprehensively investigate changes in physical performance across different habitual PA levels during the menopausal transition. This study is a part of a large cohort study that, applying careful characterizations of menopausal status, is designed to capture changes that occur in women during menopause. The narrow age range of the present participants rendered the effect of chronological aging on physical performance minor in comparison with the effect of reproductive aging. Furthermore, our study employed comprehensive measurements of physical performance to capture different dimensions of strength (dynamic, isometric, endurance, power) which may vary according to the underlying physiology and be differentially influenced by changes in hormonal status.

## Conclusions

The results of this longitudinal study showed that both early and late perimenopausal women show declines in physical performance during the transition to postmenopause. The decline is more pronounced for muscle strength and power than for walking performance. While women with high PA showed an increase in handgrip strength after the menopausal transition, they showed a greater decline than the medium, low, or inactive physically active groups in lower limb muscle strength and muscle power. Interventional studies are needed to establish whether PA benefits women during the menopausal transition.

## Supplementary Material

glaa292_suppl_Supplementary_Table_1

glaa292_suppl_Supplementary_Table_2
