# Supplementary material for: Physical Performance During the Menopausal Transition and the Role of Physical Activity
Source: J Gerontol A Biol Sci Med Sci. 2020 Nov 24;76(9):1587–90. doi: 10.1093/gerona/glaa292 (PMC8361353; doi:10.1093/gerona/glaa292)
Supplement: glaa292_suppl_Supplementary_Table_1 [file glaa292_suppl_supplementary_table_1.docx]

# Supplementary Table 1. Changes in physical performance during the menopausal transition in the early- and late perimenopausal groups (excluding HT and progestogen users)

| Variables,  group, *n* | Time | |  | Change, % (95 % CI) | ANOVA, *p-*values | | | | |  |
| --- | --- | --- | --- | --- | --- | --- | --- | --- | --- | --- |
|  | Baseline, mean  (95% CI) | Post, mean  (95% CI) |  |  |  |  |  |  |  |  |
|  |  |  |  |  | time | | group | | time×group |  |
| **Hand grip (N)** | | | |  |  | |  | |  |  |
| Total sample, *n*=188 | 310 (302; 318) | 304 (295; 312) |  | **-1.5 (-2.8; -0.2**) | **0.003** | | 0.659 | | 0.066 |  |
| Early peri*, n*=76 | 317 (304; 330) | 305 (292; 318) |  | **-3.2** (**-6.0; -0.5**) |  | |  | |  |  |
| Late peri, *n*=112 | 306 (295;315) | 3034 (292; 314) |  | -0.5 (-2.8; 1.9) |  |  |  |  |  |  |
| **Maximum knee extension torque (Nm)** | | | |  |  | |  | |  |  |
| Total sample, 169 | 155 (150; 160) | 150 (145; 155) |  | **-2.6** (**-4.1; -1.3**) | **<0.001** | | 0.894 | | 0. 321 |  |
| Early peri, *n*=65 | 155 (148; 163) | 148 (140; 157) |  | **-4.4** (**-7.1; -1.7**) |  | |  | |  |  |
| Late peri, *n*=104 | 155 (148; 161) | 151 (144; 157) |  | -1.6 (-4.3; 1.1) |  |  |  |  |  |  |
| **Vertical jumping height (cm)** | | | |  |  | |  | |  |  |
| Total sample, *n*=180 | 18.8 (18.3; 19.4) | 18.3 (17.7; 18.9) |  | **-2.6** (**-3.7; -1.4**) | **<0.001** | | 0.566 | | 0.550 |  |
| Early peri*, n*=71 | 19.1 (18.2; 20.0) | 18.4 (17.5; 19.4) |  | **-3.1 (-5.7; -0.6)** |  | |  | |  |  |
| Late peri, *n*=109 | 18.7 (18.0; 19.4) | 18.2 (17.4; 18.9) |  | **-2.2 (-4.3; -0.1)** |  |  |  |  |  |  |
| **Maximum walking speed (ms^-1^)** | | | |  |  |  | |  | | |
| Total sample, *n*=191 | 2.60 (2.53; 2.67) | 2.62 (2.55; 2.68) |  | 1.2 (-0.2; 2.7) | 0.656 | | 0.681 | | 0.420 |  |
| Early peri, *n*=74 | 2.63 (2.52; 2.74) | 2.62 (2.52; 2.72) |  | 0.5 (-2.1; 3.2) |  | |  | |  |  |
| Late peri*, n*=117 | 2.59 (2.50; 2.67) | 2.61 (2.53; 2.70) |  | 1.6 (-2.3; 2.3) |  |  |  |  |  |  |
| **Six-minute walking test (m)** | | | |  |  |  | |  | | |
| Total sample, *n*=178 | 663 (654; 672) | 677 (667; 686) |  | **2.1 (1.3; 2.8)** | <**0.001** | | 0.222 | | 0.118 |  |
| Early peri*. n*= 70 | 673 (659; 687) | 681 (666; 696) |  | **1.3 (0.1; 2.5)** |  | |  | |  |  |
| Late peri*, n*= 108 | 657 (646; 669) | 674 (662; 686) |  | **2.6 (1.6; 3.5)** |  |  |  |  |  |  |

Notes: CI = confidence interval;

Values in bold indicate statistically significant results
