# Supplementary material for: Physical Performance During the Menopausal Transition and the Role of Physical Activity
Source: J Gerontol A Biol Sci Med Sci. 2020 Nov 24;76(9):1587–90. doi: 10.1093/gerona/glaa292 (PMC8361353; doi:10.1093/gerona/glaa292)
Supplement: glaa292_suppl_Supplementary_Table_2 [file glaa292_suppl_supplementary_table_2.docx]

# Supplementary Table 2. Changes in physical performance during the menopausal transition (time) as a function of physical activity (groups) excluding HT and progestogen users

| PA,  groups, *n* | Time | |  | Change, % (95 % CI) | | ANOVA, *p-*values | | | | | | | |  |
| --- | --- | --- | --- | --- | --- | --- | --- | --- | --- | --- | --- | --- | --- | --- |
|  | Baseline, mean  (95% CI) | Post, mean  (95% CI) | |  |  |  | | | | | | | | |
|  |  |  |  |  |  | time | | | group | | | time×groups | | |
| **Hand grip (N)** | | | |  | | 0.69 | | **0.002** | | | **<0.001** | | |  |
| Inactive, *n*=24 | 278 (255; 301) | 284 (269; 298) | |  | 5.2 (-1.9; 12.3) | |  | | | | | | | |
| Low PA, n=46 | 304 (290; 319) | 299 (282; 315) | |  | -1.4 (-5.2; 2.4) | |  |  |  |  |  |  |  |  |
| Medium PA, n=95 | 310 (298; 322) | 293 (280; 205) | |  | **-5.4 (-7.8; -3.0)** | |  |  |  |  |  |  |  |  |
| High PA, n=29 | 331 (310: 352) | 343 (320; 367) | |  | **4.0 (0.2; 7.8)** | |  |  |  |  |  |  |  |  |
| **Maximum knee extension torque (Nm)** | | | |  | | **0.007** | | 0.445 | | | 0.315 | | |  |
| Inactive, *n*=18 | 149 (132; 165) | 144 (131; 158) | |  | -0.9 (-10.1; 8.4) | |  | |  | | |  | | |
| Low PA*, n*=39 | 145 (136; 155) | 145 (136; 154) | |  | 0.5 (-2.6; 3.8) | |  | |  | | |  | | |
| Medium PA, *n*=84 | 155 (149; 163) | 151 (144; 158) | |  | **-2.7 (-5.5; 0.1**) | |  | |  | | |  | | |
| High PA, n=23 | 159 (147: 171) | 150 (136; 164) | |  | **-5.5 (-9.8; -1.2)** | |  | |  | | |  | | |
| **Vertical jumping height (cm)** | | | | | | **0.022** | | | | **<0.001** | | | 0.193 |  |
| Inactive, *n*=18 | 16.5 (15.2; 18.0) | 16.6 (15.0;18.2) |  | 0.46 (-5.8; 6.7) | |  | |  | | |  | | |  |
| Low PA*, n*=42 | 18.5 (17.5; 19.5) | 18.3 (17.2; 19.5) |  | -0.7 (-4.0; 2.7) | |  | |  | | |  | | |  |
| Medium PA, *n*=86 | 18.5 (17.7; 19.3) | 17.7 (16.9; 18.5) |  | **-4.1 (-6.1; -2.0**) | |  | |  | | |  | | |  |
| High PA, n=28 | 21.4 (19.9: 23.0) | 20.8 (19.2; 22.4) |  | **-**2.8 (-6.6; 1.13) | |  | |  | | |  | | |  |
| **Maximum walking speed (ms^-1^)** | | |  |  | | 0.152 | | 0.168 | | | 0.926 | | |  |
| Inactive, n=21 | 2.54 (2.34; 2.73) | 2.61 (2.43; 2.79) |  | 3.0 (-0.2; 6.3) | |  | |  | | |  | | |  |
| Low PA, n=24 | 2.61 (2.48; 2.74) | 2.63 (2.51; 2.75) |  | 1.4 (-1.2; 4.1) | |  | |  | | |  | | |  |
| Medium PA, n=90 | 2.58 (2.47; 2.68) | 2.60 (2.51; 2.70) |  | 1.8 (-0.5; 4.1) | |  | |  | | |  | | |  |
| High PA, n=30 | 2.78 (2.61: 2.96) | 2.80 (2.64; 2.96) |  | 1.3 (-1.9; 4.5) | |  | |  | | |  | | |  |
| **Six-minute walking test (m)** | | |  |  | | **<0.001** | | **<0.001** | | | 0.427 | | |  |
| Inactive, n=19 | 641 (618; 664) | 637 (624; 670) |  | 1.1 (-1.3; 3.6) | |  | |  | | |  | | |  |
| Low PA, n=43 | 649 (633; 665) | 666 (651; 681) |  | **2.7 (1.4; 4.0)** | |  | |  | | |  | | |  |
| Medium PA, n=85 | 660 (647; 672) | 677 (662; 692) |  | **2.6 (1.6; 3.7)** | |  | |  | | |  | | |  |
| High PA, n=26 | 700 (677: 722) | 708 (685; 732) |  | 1.3 (-0.5; 3.1) | |  | |  | | |  | | |  |

Notes: CI = confidence interval;

All analyses adjusted for the duration of HT use;

Values in bold indicate statistically significant results.
